# Comparison of ELF, FibroTest and FibroScan for the non-invasive assessment of liver fibrosis

**DOI:** 10.1186/1471-230X-10-103

**Published:** 2010-09-09

**Authors:** Mireen Friedrich-Rust, William Rosenberg, Julie Parkes, Eva Herrmann, Stefan Zeuzem, Christoph Sarrazin

**Affiliations:** 1 Department of Internal Medicine 1, J.W. Goethe-University Hospital, Frankfurt, Germany; 2 Centre for Hepatology, Division of Medicine, University College London, London, UK; 3 Department of Public Health and Medical Statistics, University of Southampton, Southampton, UK; 4 Institute of Biostatistics and Mathematical Modelling, Faculty of Medicine, J.W. Goethe-University, Frankfurt

## Abstract

**Background:**

FibroTest (FT) is the most frequently used serum fibrosis marker and consists of an algorithm of five fibrosis markers (alfa2-macroglobulin, apolipoproteinA1, haptoglobin, GGT, bilirubin). The Enhanced Liver Fibrosis (ELF) test consists of an algorithm of three fibrosis markers (hyaluronic acid, amino-terminal propeptide-of-type-III-collagen, tissue-inhibitor of matrix-metaloproteinase-1). While a systematic review has shown comparable results for both individual markers, there has been no direct comparison of both markers.

**Methods:**

In the present study, the ELF-test was analyzed retrospectively in patients with chronic liver disease, who received a liver biopsy, transient elastography (TE) and the FibroTest using histology as the reference method. Histology was classified according to METAVIR and the Ludwig's classification (F0-F4) for patients with chronic hepatitis C and B virus (HCV, HBV) infection and primary biliary cirrhosis (PBC), respectively.

**Results:**

Seventy-four patients were analysed: 36 with HCV, 10 with HBV, and 28 with PBC. The accuracy (AUROC) for the diagnosis of significant fibrosis (F≥2) for ELF and FibroTest was 0.78 (95%CI:0.67-0.89) and 0.69 (95%-CI:0.57-0.82), respectively (difference not statistically significant, n.s.). The AUROC for the diagnosis of liver cirrhosis was 0.92 (95%CI:0.83-1,00), and 0.91 (95%CI:0.83-0.99), respectively (n.s.). For 66 patients with reliable TE measurements the AUROC for the diagnosis of significant fibrosis (cirrhosis) for TE, ELF and FT were 0.80 (0.94), 0.76 (0.92), and 0.67 (0.91), respectively (n.s.).

**Conclusion:**

FibroTest and ELF can be performed with comparable diagnostic accuracy for the non-invasive staging of liver fibrosis. Serum tests are informative in a higher proportion of patients than transient elastography.

## Background

For different causes of chronic liver disease assessment of liver fibrosis is important to estimate the prognosis and to determine surveillance strategies for liver cancer. In addition, for chronic viral hepatitis the degree of liver fibrosis is one important parameter for decision on antiviral therapy [1]. At present, liver biopsy is still most commonly used as reference standard for the assessment of liver fibrosis. However, it is an invasive method associated with patient discomfort and in rare cases with serious complications [2]. In addition, the accuracy of liver biopsy is limited due to sampling error and significant intra- and inter-observer variability in histological staging [3,4]. Therefore, research has focused on the evaluation of non-invasive methods for the assessment of liver fibrosis. Transient elastography (FibroScan, Echosens, France, [TE]) [5,6] and the serological fibrosis marker FibroTest (Biopredictive, France, [FT]) [7,8] have been evaluated most frequently. FibroTest consists of an algorithm of five fibrosis markers (alfa2-macroglobulin, apolipoproteinA1, haptoglobin, GGT, bilirubin). The Enhanced Liver Fibrosis Test (ELF, Siemens Diagnostics) [9,10] consists of an algorithm of three fibrosis markers (hyaluronic acid, amino-terminal propeptide of type III collagen, tissue inhibitor of matrix metaloproteinase 1). The aim of the present study was to analyze the ELF test using frozen serum samples from patients with chronic liver disease that received a liver biopsy, transient elastography (TE) and the FibroTest and to compare the results of the non-invasive tests using histology as reference method.

## Methods

The study period for sample acquisition was from September 2005 to June 2008. The Serum bank of the J.W. Goethe-University Hospital was checked for serum of patients with chronic liver disease, who received a liver biopsy, transient elastography and FibroTest. The patients were included in the present study, if frozen serum was available dated around the time of the performance of FibroTest. The time interval between sample acquisition for the FibroTest and ELF was up to one week without any therapeutic interventions between the performances of the two tests. As the mean progression rate of liver fibrosis in untreated patients was estimated 0.085-0.120 fibrosis stages on the Metavir scoring system per year [11] a time interval between liver biopsy and the performance of the non-invasive methods of up to 12 months was accepted for enrollment in the present study. The time interval between liver biopsy and study inclusion ranged from 0 to 10 months (mean 10 ± 10 weeks, median 3 weeks). The indication for liver biopsy was the determination of histological fibrosis and inflammation. Written informed consents were obtained from all patients and the study was conducted in agreement with the Declaration of Helsinki and Good Clinical Practice guidelines (ethics committee of Johann Wolfgang Goethe-University).

### Liver Histology

Liver biopsy specimens were fixed in 4%-buffered formalin and embedded in paraffin. Two-micrometer-thick sections were stained with hematoxylin-eosin, Perls-iron-stain, dPAS (periodic-acid-Schiff after digestion with diastase), Masson-Trichrome. All biopsy specimens were analyzed by an experienced pathologist blinded to the clinical results of the patients. Liver fibrosis stages were evaluated semi-quantitatively according to the Metavir scoring-system [12] for patients infected with chronic hepatitis B or C. Liver fibrosis was staged on a F0-F4 scale: F0-no fibrosis, F1-portal fibrosis without septa, F2- portal fibrosis with few septa, F3- numerous septa without cirrhosis, F4-cirrhosis. In patients with PBC histological-stage was determined according to the Ludwig's classification[13]: stage I = inflammation and/or abnormal connective tissue is confined to portal triads; stage II = the number of normal bile ducts is reduced, the inflammation and/or fibrosis is confined to portal and periportal areas; stage III = fibrous septa link adjacent portal triads (bridging fibrosis); stage IV = cirrhosis with regenerative nodules. No stage 0 = no inflammation/fibrosis exists in the Ludwig's classification, since stage I is part of the diagnosis of PBC. The biopsies were judged as adequate, if the number of portal tracts was at least 6 and the length of liver biopsy at least 1 cm. The mean length of the included liver biopsies was 22.3 ± 9.3 mm (median 20 mm, range 10-54 mm).

### Blood Markers

The following blood parameters were determined after overnight fasting in the same laboratory on the same day as transient elastography in all patients: aspartate aminotransaminase (AST), alanine aminotransaminase (ALT), γ-glutamyl transpeptidase, alkaline phosphatase, total bilirubin, platelet count, α2-macroglobulin, apolipoprotein A1, and haptoglobin. Enzymatic activity was measured at 37°C according to International Federation of Clinical Chemistry standards.

The laboratory followed the pre-analytical and analytical recommendations required to obtain the fibrosis marker score FibroTest^® ^(Biopredictive, France) [14]. The FibroTest was computed on the Biopredictive website http://www.biopredictive.com. The security algorithms on the industrial website permitting to exclude patients with high risk profile of false positive/negative were respected [15].

Frozen serum of the above patients taken around the same time (stored at -80°C) was send to an independent reference laboratory (iQur Limited, Southampton, UK). Serum samples were analyzed for levels of tissue inhibitor of matrix metalloproteinase 1 (TIMP-1), hyaluronic acid (HA), and amino-terminal propeptide of type III collagen (P3NP) using the proprietary assays developed for ELF test by Siemens Healthcare Diagnostics Inc. (Tarrytown, New York USA). Assays were performed on an Immuno-1 auto-analyser (Siemens Healthcare Diagnostics Inc, Tarrytown, New York, USA). Results were entered into the established algorithm [10] and expressed as discriminant scores (DS) = -7.412 + (ln(HA)*0.681) + (ln(P3NP)*0.775) + (ln(TIMP1)*0.494) +10 for comparison to Metavir and Ludwig's histological staging.

### Transient Elastography

Transient Elastography (TE) was performed using FibroScan^® ^(Echosens, France). This machine is equipped with a probe including an ultrasonic transducer mounted on the axis of a vibrator. A vibration transmitted from the vibrator towards the tissue induces an elastic shear wave that propagates through the tissue. These propagations are followed by pulse-echo ultrasound acquisitions and their velocity is measured which is directly related to tissue stiffness. Results are expressed in kilopascal. Details have been described in previous studies [16]. The examination was performed on the right lobe of the liver through the intercostal space. After the area of measurement was located, the examiner pressed the button of the probe to start the acquisition. The measurement depth was between 25 and 65 mm. As suggested by the manufacturer ten successful acquisitions were performed on each patient. Only TE-results obtained with 10 valid measurements, with a success-rate of at least 60% and an interquartile range ≤30% were considered reliable. FibroScan failure is defined when less than 10 valid measurements are obtained.

### Statistical Analysis

Statistical analysis was performed using BiAS for Windows (version 9.04, epsilon 2009, Frankfurt, Germany). Correlations were assessed by Spearmans correlation coefficient. The diagnostic performance of ELF, FibroTest and TE was assessed using receiver-operating-characteristic (ROC)-curves. The ROC-curve represents a plot of sensitivity versus 1-specificity for all possible cut-off values for prediction of the different fibrosis stages, respectively. The areas-under-the-ROC-curves (AUROC) as well as 95%-CI of AUROC were calculated. AUROC values for different diagnostic criteria for the same data set were compared with the non-parametric DeLong-test. Note that AUROC values for the different methods are correlated and that this test accounts for such correlations. Therefore, it may find significant differences in diagnostic accuracy even when confidence intervals of the single AUROC values, which ignore these correlations, are overlapping. Since two different fibrosis staging systems (Metavir and Ludwig's) were used to classify histology, and both systems use scores ranging from 0 to 4, the scoring systems were pooled for the overall calculation of the mean-AUROC. In case of diagnosing fibrosis stages greater than or equal 2 versus stages less than 2, we also calculated the differences between mean advanced, versus mean non-advanced fibrosis stages (DANA)-adjusted AUROC according to Poynard et al. [17] for a standardized DANA value of 2.5. Note that this adjustment was only validated for HCV patients and for FibroTest only. Assuming that, using other methods (TE and ELF) and in other pathologies, the spectrum bias has the same profile as for FT in HCV, we extrapolated the algorithm to adjust all the AUROCs in the present study.

Using cut-off values defined for the prediction of fibrosis in previous studies, sensitivity, specificity, positive- and negative-predictive-values positive- and negative likelihood ratios were calculated for the present study.

## Results

Seventy-four patients were included in the analysis: 36 patients with HCV infection, 10 with HBV infection, and 28 with PBC. Patients' characteristics are shown in table 1.

**Table 1:** Patient's characteristics

Characteristics	Patients (n = 74)
Sex:	31 male/43 female patients
Age:	mean±SD: 50 ± 13 years, median 50 years, range: 18-77 years
BMI:	mean±SD: 25 ± 5 kg/m2, median: 25 kg/m2, range 17-36 kg/m2
AST:	mean±SD: 42 ± 32 IU/L, median 31 IU/L, range: 21 - 207 IU/L
ALT:	mean±SD: 56 ± 67 IU/L, median: 35.5 IU/L, range: 7 - 460 IU/L
GGT:	mean±SD: 66 ± 79 IU/L, median: 39.5 IU/L, range 8 - 459 IU/L
Total bilirubin:	mean±SD: 0.71 ± 0.42 mg/dL, median: 0.6 mg/dL, range: 0.2 - 2.4 mg/dL
Platelet count:	mean±SD: 214 ± 75 × 103/mm3, median 226 × 103/mm3, range: 22-351 × 103/mm3
Histological Fibrosis stage	
F0	4 patients
F1	21 patients
F2	18 patients
F3	20 patients
F4	11 patients

SD = standard deviation, pat. = patients; ULN = upper limit of normal. BMI = body mass index. AST = aspartate aminotransaminase. ALT = alanine aminotransaminase. GGT = gamma-glutamyl transpeptidase. Normal value ALT and AST: female <35 U/l, male: <50 U/l; GGT female: <39 U/l, male: <66 U/l

The Spearman correlation coefficient of FibroTest and ELF with the different histological stages were 0.44, and 0.61, respectively (all p < 0.0001). The correlation coefficient between FibroTest and ELF was 0.62 (p < 0.0001).

**Figure 1:**
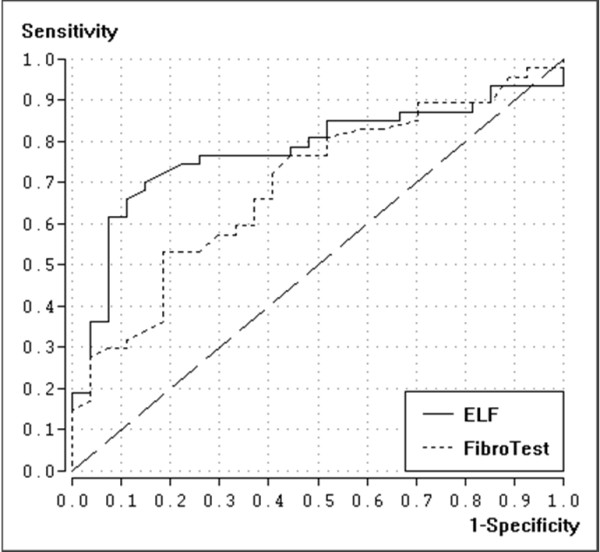
Receiver-operating characteristic (ROC) curves for FibroTest and ELF for diagnosis of significant fibrosis (F ≥ 2)

**Figure 2:**
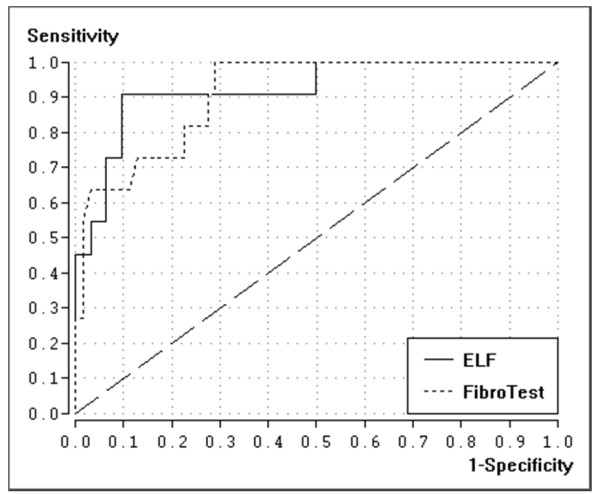
Receiver-operating characteristic (ROC) curves for FibroTest and ELF for diagnosis of liver cirrhosis (F = 4)

The diagnostic accuracy (AUROC) for the diagnosis of significant fibrosis (F≥2) for ELF and FibroTest was 0.78 (95%-CI: 0.67-0.89) and 0.69 (95%-CI: 0.57-0.82), respectively (s. figure 1). The difference was not statistically significant (p = 0.20). The AUROC for the diagnosis of severe fibrosis (F≥3) was 0.79 (95%-CI: 0.67-0.91) and 0.72 (95%-CI: 0.60-0.84), respectively (p = 0.22). The AUROC for the diagnosis of liver cirrhosis was 0.92 (95%-CI: 0.83-1.00) and 0.91 (95%-CI: 0.82-0.99), respectively (p = 0.91) (s. figure 2). Details are shown in table 2.

**Table 2:** Area under ROC-curve (95% Confidence intervall) for transient elastography, FibroTest, and ELF according to Metavir/Ludwig's fibrosis stage

	Method	F ≥ 2 (F2,3,4)	F ≥ 2 adjusted*^1^	F ≥ 3	F = 4
n = 74	FibroTest	0.69 (0.57 - 0.82)	0.74 (0.62 - 0.87)	0.72 (0.60 - 0.84)	0.91 (0.83 - 0.99)
	ELF	0.78 (0.67 - 0.89)	0.83 (0.72 - 0.94)	0.79 (0.67 - 0.91)	0.92 (0.83 - 1.00)

n = 66	Transient Elastography	0.80 (0.69 - 0.91)	0.85 (0.74 - 0.96)	0.66 (0.51 - 0.82)	0.94 (0.86 - 1.00)
	FibroTest	0.67 (0.54 - 0.80)	0.72 (0.59 - 0.85)	0.63 (0.48 - 0.79)	0.91 (0.82 - 0.99)
	ELF	0.76 (0.64 - 0.88)	0.81 (0.69 - 0.93)	0.69 (0.54 - 0.85)	0.92 (0.83 - 1.00)

*^1^Dana-adjusted AUROC according to Poynard et al.[17] for a uniform DANA of 2.5

Eight patients were excluded because of unreliable TE-measurements (less than 10 valid measurements, success rate <60%, or IQR >30%). Therefore only 66 patients were available for the comparison of TE with FT and ELF. For these 66 patients the Spearman correlation coefficient between TE, FibroTest and ELF with the different histological stages were 0.58, 0.42, and 0.58, respectively (p < 0.001). The correlation coefficient between TE and FibroTest was 0.67 (p < 0.0001), between TE and ELF 0.65 (p < 0.0001), and between FibroTest and ELF 0.62 (p < 0.0001), respectively. For these 66 patients the AUROC for the diagnosis of significant fibrosis for TE, ELF and FT was 0.80, 0.76, and 0.67, respectively (p = 0.42 for TE vs ELF, p = 0.08 for TE vs FT, p = 0.23 for ELF vs FT). The AUROC for the diagnosis of severe fibrosis (F≥3) was 0.66, 0.69 and 0.63, respectively (p = 0.60 for TE vs ELF, p = 0.68 for TE vs FT, p = 0.35 for ELF vs FT). And the AUROC for the diagnosis of liver cirrhosis was 0.94, 0.92, and 0.91, respectively (p = 0.60 for TE vs ELF, p = 0.42 for TE vs FT, p = 0.66 for ELF vs FT). Details are shown in table 2.

The diagnostic performance of FibroTest, ELF and transient elastography in the prediction of significant fibrosis (F≥2), severe fibrosis (F≥3), and cirrhosis using cut-offs defined in previous studies are shown in table 3.

**Table 3:** Diagnostic performance of FibroTest, ELF and Transient Elastography in the prediction of significant fibrosis (F≥2), severe fibrosis (F≥3), and cirrhosis for all patients, for HCV patients only, and for PBC patients only

Test	Diagnosis	Fibrosis Stage	Cut-off	Sensitivity	Specificity	PPV	NPV	Positive LR	Negative LR
**FibroTest**	All patients [7]	F ≥ 2	0.32	0.57	0.60	0.74	0.42	1.43	0.71
		F ≥ 3	0.59	0.39	0.88	0.71	0.67	3.33	0.69
		F = 4	0.73	0.64	0.90	0.54	0.93	6.68	0.40
	HCV only [7]	F ≥ 2	0.32	0.81	0.60	0.84	0.55	2.02	0.32
		F ≥ 3	0.59	0.65	0.79	0.73	0.71	3.07	0.45
		F = 4	0.73	0.67	0.81	0.54	0.88	3.60	0.41
	PBC only [7]	F ≥ 2	0.32	0.30	0.75	0.75	0.30	1.20	0.93
		F ≥ 3	0.59	0.08	1.00	1.00	0.56	invalid	0.92
		F = 4	0.73	0.00	0.96	0.00	0.96	0.00	1.04

**ELF**	All patients [9,18]	F ≥ 2	* 9.78	0.78	0.80	0.88	0.65	3.88	0.28
		F ≥ 3	* 10.22	0.74	0.70	0.64	0.79	2.45	0.37
		F = 4	* 10.31	0.91	0.62	0.29	0.98	2.39	0.15
	HCV only [9]	F ≥ 2	9.78	0.85	0.80	0.92	0.67	4.23	0.19
		F ≥ 3	10.22	0.82	0.74	0.74	0.82	3.13	0.24
		F = 4	10.31	0.89	0.63	0.44	0.94	2.40	0.18
	PBC only [22]	F ≥ 2	9.69	0.65	0.75	0.87	0.46	2.60	0.47
		F ≥ 3	9.71	0.62	0.53	0.53	0.62	1.32	0.72
		F = 4	9.89	1.00	0.52	0.07	1.00	2.01	0.00

**TE**	All patients [32-34]	F ≥ 2	* 7.20 kPa	0.64	0.76	0.85	0.50	2.71	0.47
		F ≥ 3	* 12.50 kPa	0.50	0.87	0.74	0.70	3.80	0.58
		F = 4	* 17.60 kPa	0.82	0.91	0.64	0.96	9.00	0.20
	HCV only [33]	F ≥ 2	7.10 kPa	0.79	0.50	0.79	0.50	1.58	0.42
		F ≥ 3	9.50 kPa	0.67	0.79	0.71	0.75	3.17	0.42
		F = 4	12.50 kPa	0.78	0.84	0.64	0.91	4.86	0.27
	PBC only [34]	F ≥ 2	7.30 kPa	0.39	1.00	1.00	0.31	invalid	0.61
		F ≥ 3	9.80 kPa	0.25	0.91	0.75	0.53	2.75	0.83
		F = 4	17.30 kPa	1.00	0.95	0.50	1.00	22.00	0.00

The cut-offs were evaluated in previous studies, the references are cited in the diagnosis column in brackets for all patients, for HCV, and PBC respectively and can be found in the reference list. For all patients the individual cut-offs for HCV and PBC were used if available, and for HBV the cut-offs of TE were used from a study [32] evaluating patients with different chronic liver diseases, and of ELF the cut-offs of HCV [9] were used. For FibroTest no cut-offs for PBC have been evaluated, the cut-offs for viral hepatitis [7] were used, respectively

TE = transient elastography, PPV = positive predictive value, NPV = negative predictive value, LR = likelihood ratio

## Discussion

At present, transient elastography and the serum marker FibroTest are the most intensively evaluated non-invasive methods for the assessment of liver fibrosis. The results of the present study for transient elastography, FibroTest and ELF are in accordance with the results of previous studies [5-8,10,18]. However, the latter is of more clinical importance for estimation of prognosis, surveillance and treatment decisions before patients develop liver cirrhosis [1]. Furthermore inter and intra-observer discrepancies in histological classification of lesser stages of fibrosis are more prevalent than for higher stages and this may account for the observed under performance of non-invasive tests when compared to histology as a reference standard.

A systematic review has shown comparable results for ELF and FibroTest but no direct comparison of both markers had been performed before [19]. This is the first study, comparing transient elastography, FibroTest and ELF in the same study population. The results of the three non-invasive methods using different approaches were comparable for the diagnosis of significant fibrosis and cirrhosis in the present study.

The ELF test was developed in an international multicenter cohort study with 1021 subjects with chronic liver disease using discriminant analysis to identify the above mentioned algorithm having investigated specific markers of matrix turnover as well as indirect markers of liver function [10]. The ELF test was validated in Non-Alcoholic Liver Disease in adults [20] and in children [21], in PBC and hepatitis C [9,22]

One study [23] has compared the FibroTest with ELF in a subgroup of patients infected with chronic hepatitis C. In this study, instead of using the ELF assays for HA, PIIINP and TIMP-1 that have been developed by Siemens Healthcare Diagnostics specifically for use in the ELF test; and instead of performing the assays on the Immuno-1 auto-analyser on which the ELF test is currently validated and CE marked, the investigators used alternative assays and performed manual testing. Thus the method used by Cales et al. to measure the analytes prior to incorporating results into the ELF algorithm cannot be considered to be analogous with the present study.

In the study of Cales et al. Fibrometer, an algorithm of direct and indirect markers was evaluated and compared with other non-invasive markers in a validation group. The authors suggest that the combination of both markers might increase certain advantages and limit other disadvantages. However, no significant difference was found between the Fibrometer, FibroTest and ELF as performed by the authors in this study. Similar results were found comparing ELF and TE in 80 patients with hepatitis C with the same diagnostic accuracy for both methods (AUROC 0.91, 0.90 for cirrhosis and 0.82 for >stage 2 fibrosis) [24].

Recently, a series of algorithms based on sequential combination of non-invasive serum markers showed 93-95% accuracy in the detection or exclusion of significant liver fibrosis and a reduction of 50% of liver biopsies in this subset of patients with HCV infection [25]. However, the combination of FibroTest and ELF has not been evaluated yet. Further studies are needed to evaluate the advantages and disadvantages of both markers and in which situations they may substitute each other.

Using liver biopsy as a reference standard for the evaluation of non-invasive methods and markers has methodological limitations which may influence the performance of these tests. The accuracy of liver biopsy is limited due to intra- and inter-observer variability and sampling errors [3]. In a study on more than 10,000 virtual biopsies Bedossa et al. [3] showed that liver fibrosis stage is correctly diagnosed in only 65% of cases, if the biopsy is at least 15 mm long, in 75% if it is at least 25 mm long and, that the optimal size should be 40 mm. However, most biopsies even at specialist Hepatology centers do not fulfill these optimal criteria [26].

Data analyzing the discordance of liver biopsy and the panel marker FibroTest showed that this discordance was highly attributable to biopsy in 5% and to the panel marker in 2% (p = 0.03) [26]. The authors concluded that these shortcomings of liver biopsy lead to underestimation of the diagnostic accuracy of non-invasive markers. A recent study has demonstrated that error in the liver biopsy result itself makes it impossible to distinguish a perfect non-invasive marker from less valid assays [27]. This supports the assumption that non-invasive markers might be underestimated using liver biopsy as reference method. The ultimate validation of liver fibrosis as a marker of liver injury is its prognostic value in terms of morbidity and mortality. In a study in patients infected with chronic hepatitis C, FibroTest was shown to have a 5-year prognostic value similar to that of liver biopsy [15]. In addition, the FibroTest was shown to accurately define 4-year prognosis in patients infected with hepatitis B [28] and 10-year prognosis in patients with alcoholic liver disease [29]. A study in patients with PBC demonstrated a highly significant relationship between the baseline ELF score and the likelihood of developing a clinical complication over the next 6 years [22], which was also shown in a mixed etiology cohort of 500 patients [30].

For ultrasound based methods to measure liver fibrosis (transient elastography [FibroScan], and acoustic radiation force impulse imaging [ARFI]) long-term follow-up studies are not available yet [5,31]. Large, well-conducted randomized trials with clearly defined endpoints, i.e. assessing 5-year survival without complications related to liver disease (liver related death, liver transplantation, hepatic decompensation, variceal bleeding, hepatocellular carcinoma) are needed to compare the non-invasive methods with each other and with liver biopsy.

A limitation of the present study is its retrospective analysis and the small study population, however, this is the first study comparing an algorithm of indirect fibrosis markers (FibroTest), an algorithm of direct fibrosis markers (ELF) using the approved and validated ELF assay and analyzer and an ultrasound-based elastography method in one and the same study population. Larger prospective studies are necessary to confirm these results. Another limitation of our analysis is the inclusion of biopsies which are shorter than the usual standard of 15 mm, if at least 6 portal tracts were present. Nevertheless, the exclusion of such short biopsies had no significant effect on our results. In addition, the present study was a comparative study between the different non-invasive methods, where the quality of liver biopsy affected all methods equally.

## Conclusion

FibroTest and ELF can be performed with comparable diagnostic accuracy for the non-invasive staging of liver fibrosis.

## Competing interests

The manuscript (including the article-processing charge) is not financed by any organization. The performance of the ELF-marker was supported by the National Institute for Health Research through the University College London Hospital Comprehensive Biomedical Research Centre. William Rosenberg has been paid for providing lectures by Siemens on the topic of the ELF marker. He is named inventor on a patent wholly owned by Siemens. He has no financial or non-financial competing interests of relevance to declare. In addition, the following authors have no competing interests or funding: Mireen Friedrich-Rust, Julie Parkes, Eva Herrmann, Stefan Zeuzem, Christoph Sarrazin.

## Authors' contributions

MFR participated in the study design, data analysis and writing. WR participated in the study design, ELF testing, data analysis and writing. JP participated in the data analysis and writing. EH participated in the study design and performed the statistical analysis. SZ participated in the study design and coordination. CS conceived the study and participated in the study design and coordination. All authors collected and analyzed data, contributed to preparing the manuscript, and read and approved the final manuscript.

## Funding

none

## Pre-publication history

The pre-publication history for this paper can be accessed here:

http://www.biomedcentral.com/1471-230X/10/103/prepub
